# Geographic Distribution of Childbirth among Adolescents in Cameroon from 2003 to 2005

**DOI:** 10.1155/2010/805165

**Published:** 2010-07-27

**Authors:** P. M. Tebeu, J. D. Kemfang, D. I. Sandjong, E. Kongnyuy, G. Halle, A. S. Doh

**Affiliations:** ^1^Ligue d'Initiative et de Recherche Active pour la Santé et l'Education de la Femme (LIRASEF), Cameroon; ^2^Department of obstetrics and Genecology, University Teaching Hospital, Yaoundé, Cameroon; ^3^Child and Reproductive Health Group, Liverpool School of Tropical Medicine, Liverpool L3 5Q A, UK

## Abstract

*Objective*. To determine the frequency and the trend of adolescents (10–19 years) in childbirth within a period of 3 years in referral maternity units in Cameroon. *Method*. Descriptive and retrospective study for a 3-year period (2003–2005) in referral maternity units headed by a qualified Obstetrician-Gynecologist. We analyzed the trend and geographic distribution of 8222 adolescent deliveries over 3 years. Epi Info 3.5 software was used for data analysis. Chi square test for trend was used to assess the contribution of adolescent deliveries over years. The trend was considered significant if *P* < .05. *Results*. During the period of the study, there was a total of 8387 deliveries. We excluded 165 women because of lack of information about age. We therefore included a total of 8222 adolescent deliveries. Overall, the contribution of adolescents to deliveries ranged from 6.87% to 26.51%, depending on the region with a national mean of 14.23%. Adolescents aged 16 or less contributed to 2.82% of deliveries while those aged from 17 to 19 contributed to 11.41%. The contribution of adolescents to deliveries decreased significantly over 3 years (*P* < .0001). *Conclusion*. The study underscores the importance of Public Health programs in strengthening maternity services for adolescents in Cameroon while taking into consideration geographic differences.

## 1. Introduction

Adolescence constitutes a period of rapid growth, both physical and emotional. It encompasses young people aged 10 to 19 years. Adolescent pregnancy is considered as high risk and is associated with high rates of obstetric and psychological complications [1–5]. One out of four pregnancy-related deaths occurs in adolescents [6]. One of the objectives of safe motherhood programs, of which family planning is an essential component, is to reduce the contribution of this age group to childbirth [7]. Contraceptive prevalence in Cameroon is low among married women and shows uneven distribution varying from 2.6% in North Cameroon to 43.9% in Yaoundé [8]. This low-contraceptive use prevalence among married couples is reported at 2.6%, 3.3%, and 17.6% in North, Far North, and Adamawa regions. The low prevalence use among married couples suggests that once a woman is married she is exposed to pregnancy irrespective of her age. 

In Cameroon, the illiteracy rate varies from 44.3% in Far North to 3.4% in Yaoundé, with a rate of 33%, 39%, and 44%, respectively, in Adamaoua, Far North and North. Illiteracy predisposes to early marriage and early childbearing among adolescents [9].

Existing data on Cameroon indicate that adolescents represent 21% of the total population and contribute for 13.8% of deliveries [9, 10]. Two recent studies revealed that adolescents' contribution to deliveries is 6.69% at the University Teaching Hospital in Yaoundé (capital city of Cameroon) and 26.54% in Maroua Regional Hospital in the Far North Region [11, 12]. These findings suggest that there is a great disparity in the geographic distribution of adolescent deliveries in Cameroon. Knowledge of this geographic distribution can provide useful information for updating and strengthening adolescent reproductive health strategies in Cameroon.

**Objective**

To determine the frequency and the trend of adolescents (10–19 years) in childbirth within a period of 3 years in referral maternity units in Cameroon.

## 2. Methods

### 2.1. Study Design and Setting

It is a retrospective study carried out over a period of 3 years to assess adolescents' contribution to deliveries in referral maternity units in Cameroon. 

**Figure 1:**
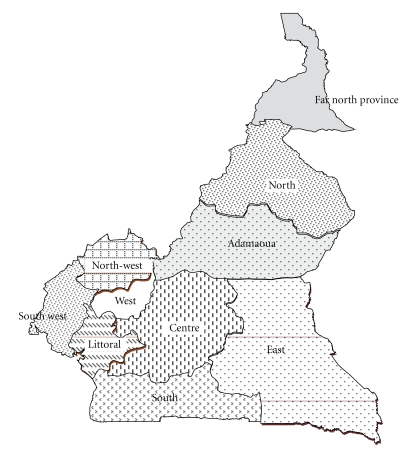
Regional presentation of Cameroon.

We included referral maternity units supervised by Obstetrician-Gynecologist in the 10 regions of Cameroon. We identified referral maternity units by region: Centre (General Hospital, Gynaeco-Obstetric and Pediatric Hospital, University Hospital Centre, Yaoundé Central Hospital), Littoral (General Hospital, Laquintinie Hospital), South (Ebolowa Provincial Hospital, Nsangmelima District Hospital), West (Bafoussam Provincial Hospital), North West (Bamenda Provincial Hospital), South West (Limbe Provincial Hospital, Assimilated Provincial Hospital of Buea), East (Bertoua Provincial Hospital), Adamaoua (Ngaoundere Provincial Hospital), North (Garoua Provincial Hospital), and Far North (Maroua Provincial Hospital). We excluded Buea and Bertoua Provincial Hospitals for insufficient data during the period of study. We therefore analyzed data of 14 referral hospitals from ten regions of the Country (Figure 1).

### 2.2. Population

The study population consisted of girls and women who were received in the delivery room of the maternity units at the referral hospitals in Cameroon during a three-year period (2003 to 2005) irrespective of where they had antenatal care.

### 2.3. Variables

We collected data retrospectively from delivery registers. We contacted at least one Obstetrician-Gynecologist in each selected maternity unit, either by telephone or by email. We explained the objectives of the study and gave them the data collection instrument. The variables specified in the instruments were name of the hospital, year of delivery (2003, 2004, and 2005), number of deliveries in the year separated by classes (the overall, less than 17 years, 17–19 years, with age nonspecified). We did not include women whose pregnancy was terminated before 28 weeks (7 months) and commonly considered as abortions. The sociodemographic variables, management of labor, and pregnancy outcome were not assessed because the objective of this study was mainly to evaluate the proportion of deliveries by adolescents.

### 2.4. Statistical Analysis

We calculated the crude rate and annual contribution of adolescents to deliveries. The maternity unit of Laquintinie Hospital reported cumulative data over a period of three years and the national annual rates reported in this study did not include this health facility. Data were analyzed with Epi Info 3.5. The Chi^2^ square test for trend was used to evaluate the contribution of adolescents to deliveries over time. The trend was considered statistically significant if *P* < .05. We grouped the towns into zones ranging from I to V according to the contribution of adolescents to deliveries. The zones were defined as follows: zone I (<10%), zone II (10–14%), zone III (15–19%), zone IV (20–25%), and zone V (≥25%).

## 3. Results

The 14 hospitals included in this study fall into several categories of the health facilities in Cameroon. Category 1 (University Teaching Hospital, Gynecology-Obstetric and Pediatric Hospital, General Hospitals of Yaoundé and Douala), Category 2 (Yaoundé Central Hospital and Laquintinie Hospital in Douala), Category 3 (Provincial Hospitals of Maroua, Garoua, Ngaoundere, Bertoua, Bamenda, Limbe and Bafoussam), and Category 4 (Nsangmelima District Hospital) (Table 1). During the study period, we had a total of 57787 births where maternal age was available. We excluded of the analysis 165 births (1.97%) where maternal age was not specified. 

Overall, we found that 8222 of the births were by teenage mothers. We found that during the study period, adolescents contributed for 12.83% to 15.08% of deliveries and this percentage decreased significantly over time (*P* for trend <.001) (Table 2). The contribution of adolescents to deliveries varies from region to region ranging from 6.87% in the Bafoussam (West) to 26.51% in Maroua (Far North) with an overall national frequency of 14.23%. The contribution of adolescents aged 16 or less varied from 0.81% in the West to 6.15% in Far North. 

We observed that Bafoussam (West) and Yaoundé (Centre) had the lowest number of adolescent deliveries as a percentage of all births, with 6.87% and 8.68%, respectively. At the central level, we found that General Hospitals had lower rates of adolescent deliveries. This is highlighted by the difference of adolescents' contribution to deliveries in Douala General Hospital (1.06%) and Laquintinie Hospital (8.36%), two hospitals in the same city. We found that 4 out of 10 towns included in this study were of zones IV and V with more than 20% of deliveries among adolescents and those four towns were Nsangmelima, Ngaoundere, Garoua, and Maroua.

## 4. Discussion

Several studies have reported deliveries among adolescents as a percentage of all births and this percentage varies from 6% to 26% [11–16]. In our study, we found that adolescents contributed to 14.23% of deliveries in referral hospitals in Cameroon. This percentage is lower than that reported in South Africa and in Maroua-Cameroon, where the authors found that one out of every four mothers was aged less than 20 years [12, 14].

Data from Far North in Cameroon revealed that 97% of adolescents are married at the time of delivery [17]. Early marriage can be considered as an indicator for early childbearing, since very few married teenagers (3.3%) take any modern contraception as shown by the 2004 DHS [8].

Another study on 8174 deliveries (which included women with all parities) in the University Hospital Centre in Yaoundé showed that adolescents contributed to 6.69% of deliveries [11]. In the present study, we found that adolescents contributed to 9.20% of deliveries in the same hospital (University Hospital Centre in Yaoundé) suggesting a rising rate of adolescent childbirth in this setting.

One study in the USA revealed that 6.21% of women giving birth were aged 15 to 19 [18]. Creatsas and Elsheikh recently reported that 7.53% of deliveries in Greece occur among adolescents [16], a rate comparable to that of Yaoundé Cameroon [11] and USA [18]. The national rate reported in our study is higher than that reported in the USA.

Our study revealed that adolescents aged 16 or less contributed to 2.82% of deliveries. This rate is comparable to the 1.21% reported previously in the University Hospital in Yaoundé, but lower than the 7.6% recently reported in Maroua-Cameroon [12, 19]. We found geographic differences in the contribution of adolescents to deliveries, with hospitals in zone IV located in the Northern regions and in Nsangmelima. The cultural and social values in northern Cameroon contribute to high fertility rate and the desire for many children. This region is dominated by Muslims and early marriage among adolescents is promoted by cultural values. 

We found that the contribution of adolescents to deliveries is decreasing over time. This observation corroborates with that of the trend in decrease of total fertility rate of the Country from 5.8 in 1991 to 5.0 in 2004 [10]. Moreover, the national modern contraceptive prevalence used among couples increased from 4% in 1991 to 14% in 2004 [8]. This suggests that multisectorial and multidisciplinary efforts to discourage early sexual debut have been effective. However, these national figures can hide isolated circumstances with increasing number of adolescents as a percentage of all births in some localities. In fact, an increasing proportion of childbirth among adolescents has previously been reported in the University Hospital Centre and was confirmed in our study.

To the best of our knowledge, this is the third among studies reported in English or French (following two previous studies in University Hospital Centre Yaoundé, and Maroua Provincial Hospital) to have analyzed the contribution of adolescents to deliveries and changes over time [11, 12]. 

The contribution of adolescents to deliveries is an indicator of early unprotected sexual activity. This does not only have demographic implications, but also affect adolescent reproductive health such as exposure to various sexually transmitted infections, HIV infection, cancer of the cervix, obstetric fistula, and death [20, 21]. 

In a recent study in the University Hospital Centre in Yaoundé, the authors reported that the first delivery among adolescents was associated with prematurity, perinea tear, fetal distress, episiotomy, use of oxytocin, delivery by cesarean section, and stillbirth [13]. Different studies have shown that adolescents, and in particular those less than 17 years, have higher rates of caesarean delivery and stillbirth [22–24]. 

Adolescents contribute significantly to deliveries in Cameroon and more especially in the Northern regions. Nowadays women have more responsibilities for themselves and for their children; they want to continue their education and have a job before childbearing. However, all women do not have access to information and sufficient education to achieve their objectives. The priority of health services and all organizations involved in the provision of adolescent reproductive health should be to reduce the contribution of adolescents to deliveries. The different risk factors for adolescent pregnancy dictate which preventive measures to take. Studies have been conducted on knowledge, attitudes, and practice among adolescents, on sexually transmitted infections and unwanted pregnancy, and it seems that the best interventions are when adolescents themselves participate in the development of the strategies [25].

Various interventions based on the introduction of modules in school curricula have been assessed with the hope to reduce unwanted pregnancy and its consequences on adolescents [26]. These interventions include school programs based on abstinence only, abstinence with information on contraception, encouragement of community activities, and teaching on competences to overcome peer pressure. 

Considering the fact that the contribution of adolescents to deliveries is higher in northern than southern Cameroon, it is essential to rethink of the strategies implemented so far in Cameroon and to undertake a study that identify specific strategies for each zone. We recommend early integration of education and reproductive health in school curriculum, and education of parents to improve parental guide of adolescents.

We acknowledge that this study has some limitations. Data collection was not easy in maternity units due to absence of registers for some years (Buea and Ebolowa) and the presence of registers with no data (Bertoua). In Cameroon, 40% of deliveries occur at home and this study concern only the women who delivered in the health facilities [27].

Additionally, some sociodemographic conditions like, educational level, marital status, illiteracy, occupation, and emotion are potential risk factors for early pregnancy; unfortunately, these factors were not assessed in the present study.

## 5. Conclusion

Adolescents contribute significantly to deliveries in Cameroon. This study highlights the need to conduct a study to determine the impact of very young age on pregnancy. Our study underscores the importance of political will and Public Health programs in strengthening adolescent health services. In particular, there is a need to readapt education strategies in order to improve health among adolescents. Despite the willingness of the state and international institutions, for any intervention to be effective, competent authorities, experts, and interested parties must come together to develop a multidisiplinary and concerted approach to reduce adolescent pregnancies. 

## Figures and Tables

**Table 1:** Characteristics of health facilities studied.

Characteristics	*N*(%)	*N* = 16
Level		
Central	6	(37.5)
Intermediate	9	(56.3)
Peripheral	1	(6,3)

Category		
Category 1	4	25.0
Category 2	2	(12.5)
Category 3	9	(56.3)
Category 4	1	(6.3)

Number of health facilities by Province		
Centre	4	(25.0)
Littoral	2	(12.5)
West	1	(6.3)
North West	1	(6.3)
South West	2	(12.5)
South	2	(12.5)
East	1	(6.3)
Adamaoua	1	(6.3)
North	1	(6.3)
Far North	1	(6.3)

Number of deliveries in 3 years		
10,000 and more	1	(6.3)
9000–9999	1	(6.3)
6000–6999	3	(18.8)
5000–5999	2	(12.5)
4000–4999	1	(6.3)
3000–3999	1	(6.3)
2000–2999	4	(25.0)
Less than 2000	3	(18.8)

*N*: Number of women who gave birth; %: Percentage.

**Table 2:** Distribution of deliveries by year during the period of the study.

Year	Deliveries per year	10–16 year		17–19 year		Total	
		*N*	(%)	*N*	(%)	*N*	(%)
2003	18554	544	(2.93)	2254	(12.15)	2798	(15.08)
2004	19687	612	(3.11)	2239	(11.37)	2851	(14.48)
2005	19546	473	(2.42)	2100	(10.74)	2508	(12.83)
2003–2005	57787	1629	(2.82)	6593	(11.41)	8222	(14.23)

Chi^2^ for trend: 30.2 and *P* = .0000; *N*  : Number of women who gave birth; %: Percentage.s
